# Dosimetry and efficiency comparison of knowledge-based and manual planning using volumetric modulated arc therapy for craniospinal irradiation

**DOI:** 10.2478/raon-2024-0018

**Published:** 2024-03-07

**Authors:** Wei-Ta Tsai, Hui-Ling Hsieh, Shih-Kai Hung, Chi-Fu Zeng, Ming-Fen Lee, Po-Hao Lin, Chia-Yi Lin, Wei-Chih Li, Wen-Yen Chiou, Tung-Hsin Wu

**Affiliations:** Department of Biomedical Imaging and Radiological Sciences, National Yang Ming Chiao Tung University, Taipei, Taiwan; Department of Radiation Oncology, Dalin Tzu Chi Hospital, Buddhist Tzu Chi Medical Foundation, Chiayi, Taiwan; School of Medicine, Tzu Chi University, Hualien, Taiwan; Departments of Radiation Oncology, Taichung Tzu Chi Hospital, Buddhist Tzu Chi Medical Foundation, Taichung, Taiwan

**Keywords:** knowledge-based planning, RapidPlan, craniospinal irradiation, volumetric modulated arc therapy

## Abstract

**Background:**

Craniospinal irradiation (CSI) poses a challenge to treatment planning due to the large target, field junction, and multiple organs at risk (OARs) involved. The aim of this study was to evaluate the performance of knowledge-based planning (KBP) in CSI by comparing original manual plans (MP), KBP RapidPlan initial plans (RP_I_), and KBP RapidPlan final plans (RP_F_), which received further re-optimization to meet the dose constraints.

**Patients and methods:**

Dose distributions in the target were evaluated in terms of coverage, mean dose, conformity index (CI), and homogeneity index (HI). The dosimetric results of OARs, planning time, and monitor unit (MU) were evaluated.

**Results:**

All MP and RP_F_ plans met the plan goals, and 89.36% of RP_I_ plans met the plan goals. The Wilcoxon tests showed comparable target coverage, CI, and HI for the MP and RP_F_ groups; however, worst plan quality was demonstrated in the RP_I_ plans than in MP and RP_F_. For the OARs, RP_F_ and RP_I_ groups had better dosimetric results than the MP group (*P* < 0.05 for optic nerves, eyes, parotid glands, and heart). The planning time was significantly reduced by the KBP from an average of 677.80 min in MP to 227.66 min (*P* < 0.05) and 307.76 min (*P* < 0.05) in RP_I_, and RP_F_, respectively. MU was not significantly different between these three groups.

**Conclusions:**

The KBP can significantly reduce planning time in CSI. Manual re-optimization after the initial KBP is recommended to enhance the plan quality.

## Introduction

Prophylactic or therapeutic craniospinal irradiation (CSI) is an option for managing certain primary brain tumors, such as medulloblastoma, or hematologic malignancies.^1^ Since the maximum field of the linear accelerator is 40 cm by 40 cm, the conventional three-dimensional conformal radiation therapy (3D-CRT) techniques for CSI use two opposed lateral craniocervical fields adjoined by two adjacent posterior spinal fields. In conventional CSI techniques, the fields are matched between the lateral and posterior fields, creating over- or underdosage within the spinal cord. To address this issue, 3D-CRT with the moving junction technique^2,3^, which involves changing different junction locations daily during the treatment course, is an option to blur the dose ununiform effect.

The moving junction technique in 3D-CRT requires the use of multiple treatment plans, which increases the complexity of treatment planning and daily treatment. Moreover, the CSI moving junction technique can only reduce the dose ununiform effect but cannot obtain dose homogeneity as a common treatment. With the development of commercial treatment planning system (TPS), volumetric modulated arc therapy (VMAT) with multi-isocenter optimization^4^ was introduced. VMAT with 360-degree beams can achieve higher conformity and better dispersion of normal organs compared to conventional 3D-CRT.^5,6^ The VMAT technique with large field overlaps for low-dose gradient junction could tolerate greater positional shifts while maintaining homogeneous dose.^7,8^ However, planning CSI using the high-precision VMAT technique is challenging and time-consuming for medical physicists due to the long treatment field from the brain to the lumbosacral region, which significantly exceeds the treatment field size of a linear accelerator and involves more than ten organs at risk. Because CSI treatment is relatively rare and only patients with possible malignancy tumor cells seeding in the craniospinal canal receive this treatment, medical physicists in many institutions are unfamiliar with this technique. The rarity of the expertise and complex planning processes make this process resource-intensive.

Knowledge-based planning (KBP) is based on a model of estimating dose-volume histograms (DVHs), which is configured by a library of historical treatment plans with the aim of improving planning efficiency.^9^ In previous studies, KBP has been adopted to treat patients with several cancer types, such as head and neck cancers and pelvic malignancies.^10,11,12,13^ KBP showed improved planning efficiency with well-reserved plan quality in those cancer sites. However, compared to those cancer sites, CSI would require more treatment isocenters and patients moving with junction feathering. Moreover, more organs at risk (OARs) needed to be considered in CSI than other treatment sites. Reviewing the literature, previous CSI studies have not compared the plan quality and cost-effectiveness of the general manual plan method and the KBP with and without re-optimization.

This study aimed to compare the plan quality and efficiency of the original manual plans (MP), KBP initial plans (RP_I_) (RapidPlan^TM^, Varian Medical Systems, Palo Alto, USA), and KBP final plans, which received further re-optimization (RP_F_) for CSI.

## Patients and methods

### Ethics statement

The Institutional Review Board of the Dalin Tzu Chi Hospital, Buddhist Tzu Chi Medical Foundation approved this study (approval number, B10804011-1) and waived the requirement for written informed consent from the patients involved because only anonymized images were retrospectively analyzed, and this study did not affect the actual treatments these patients received before.

### Patients

**FIGURE 1.**
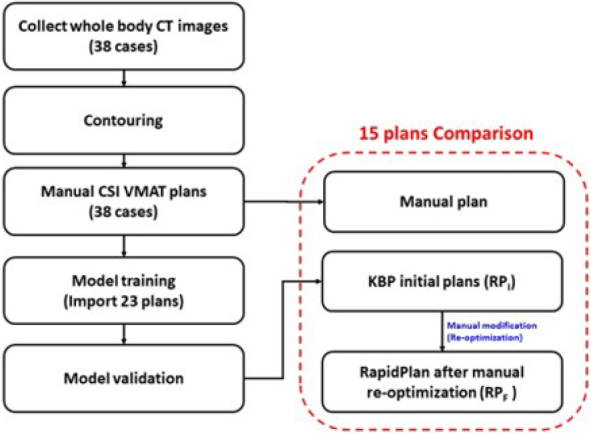
Flowchart of the study design. CSI = craniospinal irradiation; CT = computed tomography; KBP = knowledge-based planning; VMAT = volumetric modulated arc therapy

This study retrospectively collected computed tomography (CT) image sets of 38 anonymized adults assessed between 2014 and 2019. All the image sets met the requirement of immobilization, supine position, and scan from head to pelvis. The slice thickness and matrix size were 3–5 mm and 512 × 512 voxels, respectively (Figure 1).

### Target and OAR delineation

**FIGURE 2.**
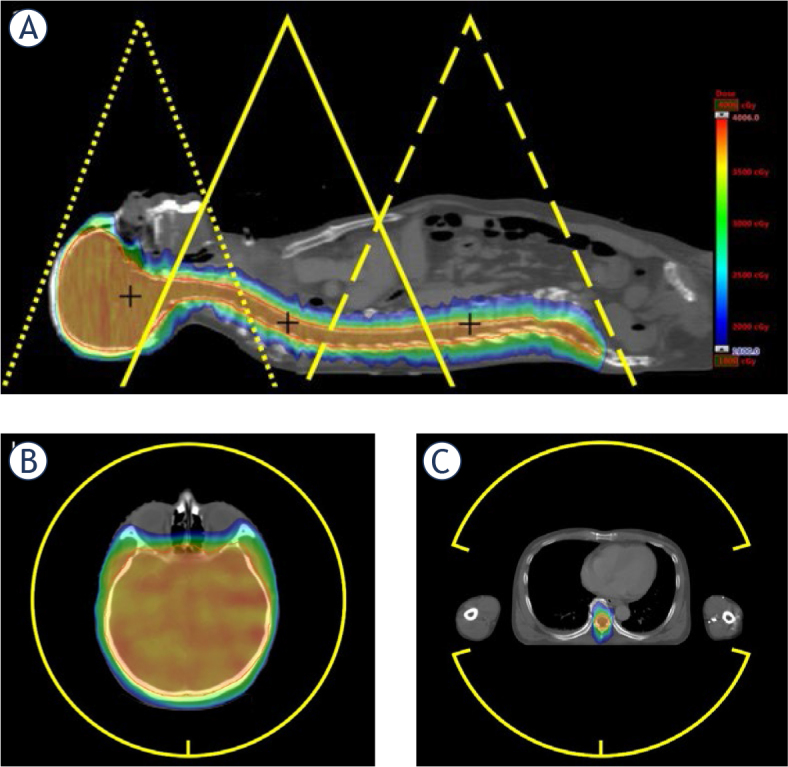
Example of the target and field setup. **(A)** The arrangement of the brain field (dotted lines), spine-superior field (solid lines), spine-inferior field (dashed lines), and their isocenters. Each field overlaps at least 5 cm for the low-dose gradient junction. **(B)** Full arc was used on the brain field. **(C)** Partial arc was used in the spinal fields for arm sparing.

The clinical target volume (CTV) includes the whole brain and spinal cord, typically extended to the lumbar spine L3 level. Assembled CTV was separated into CTV-brain, CTV-spine-superior, and CTV-spine-inferior for the multiple field optimization (Figure 2). The PTV-brain was constructed by symmetrically extending the CTV-brain by 3 mm and by adding 5 mm margin to the spine area. The maximum and minimum lengths of the CTV were 77.83 cm and 65.40 cm, while those of the PTV were 78.80 cm and 66.38 cm. The mean lengths of the CTV and PTV were 71.15 ± 4.28 cm and 72.23 ± 4.16 cm, respectively. The mean CTV and PTV were 1413.40 ± 162.18 cm^3^ and 1823.93 ± 192.14 cm^3^, respectively. For planning evaluation purposes, the PTV-brain, PTV-spine-superior, and PTV-spine-inferior were combined as PTV.

### Dose prescription

The dose prescription was 36 Gy in 18 daily fractions. All plans were normalized so that 95% of the PTV received 100% of the prescribed dose.

### Treatment planning

The 38 CT image sets of anonymized adults were imported to Eclipse TPS version 13.6 (Varian Medical Systems, Palo Alto, CA, USA). Overall, six medical physicists were participated in this study. Plans for each patient were reviewed and approved by the same physician. A TrueBeam linear accelerator (Varian Medical Systems, Palo Alto, CA, USA) equipped with a 120-leaf multileaf collimator was selected. All plans were set as 6 megavoltage for the VMAT technique. Analytical Anisotropic Algorithm dose calculation algorithm, 2.5 mm dose calculation grid, and jaw tracking were used. The mean lateral field size for the brain field is 14.76 ± 0.08 cm, while the average lateral field size for the spine field is 12.42 ± 2.52 cm. These dimensions are adjusted to encompass the entire target within a reasonable rotation range. Jaw tracking technique is used to minimize the impact of transmission leakage dose to normal organ. The collimator rotation angle is set within a range of ± 35 degrees for the head and ± 12 degrees for the spine, according to the physicist’s discretion at the time.

The whole target length was more than 100 cm, whereas the maximum single-field size of a linear accelerator at the isocenter is 40 × 40 cm. Therefore, multiple fields and three isocenters were required. The PTV-brain used two full arcs, with the isocenter positioned at the center of the brain. For the PTV-spine, two or four partial arcs were used on the PTV-spine-superior, and PTV-spine-inferior to avoid the 60–120-degree and 240–300-degree direction for arm sparing. For the sake of clinical convenience, the three isocenters were aligned along the same X-axis (left-right). The spine isocenter shared the same X and Y coordinates, differing only along the Z-axis (craniocaudal) (Figure 2).

A total of 38 MPs were generated for the 38 patients, with 23 MPs used to train the RapidPlan (RP) model, and 15 MPs used for validation and comparison (Figure 1). Using RP, 15 RP initial plans (RP_I_) were generated without manual modification, on which we performed further manual re-optimization to generate 15 RP final plans (RP_F_). Finally, we compared the following three plan groups: MP, RP_I_, and RP_F_.

### Knowledge-based planning

The RapidPlan is a commercial KBP program integrated within the Eclipse TPS. The KBP program references a library of previously clinically accepted treatment plans. It analyzes the geometric and dosimetric features, such as structure sets, field geometry, dose matrices and plan prescriptions of those plans to train a statistical model. This model is then used to predict an achievable range of DVHs and generate dose-volume objectives for a new plan.

### RapidPlan algorithm

The RapidPlan algorithm comprises two main components: model configuration and DVH estimation. The model configuration component is responsible for setting up new DVH estimation models, which are subsequently utilized in the DVH estimation component to generate estimates for an individual plan. The model configuration component encompasses two distinct phases: data extraction and model training. On the other hand, the DVH estimation component encompasses the phases of estimation generation and objective generation.

The minimum requirement of data extraction and model training was 20 plans with their targets and OARs. Among the 20 randomly selected plans for model training, the right lens of three plans were too small to evaluate. Therefore, we added three more plans to meet the training requirement.

The model training phase within the DVH estimation algorithm is dedicated to the creation of DVH estimation models. The estimation generation phase calculates for each supported structure the same metrics that were calculated during the data extraction of the DVH estimation model, except for the DVH. Once the estimation generation phase has derived the upper and lower bound DVHs, the optimization objectives placement phase translates them into optimization objectives.

### Plan quality, planning time, and monitor unit comparison

There were 27 dosimetric goals of irradiated fields and OARs were evaluated for the three groups among 15 patients. One patient had previously undergone thyroidectomy, and his thyroid dose could not be evaluated. This resulted in a total of 404 items being calculated for model evaluation. Dosimetric characteristics, such as V_95_, V_100_, V_107_, D_mean_, D_max_, and D_2_ of CTV, and PTV, were evaluated. In addition, conformity index (CI) and homogeneity index (HI) of the targets and dose gradient (R_x%_) were compared.^14^ The Radiation Therapy Oncology Group (RTOG) criteria define CI values to be between 1.0 and 2.0 in accordance with the protocol, 2.0 to 2.5 and 0.9 to 1.0 as a minor deviation, and > 2.5 and < 0.9 as a major deviation from the protocol. The CI was defined as a ratio between the volume covered by the reference isodose (36 Gy) and the target volume, as in Equation [1].

[1] $${\mathrm{CI}}_{\mathrm{RTOG}}=\frac{V_{\mathrm{RI}}}{\mathrm{TV}}$$

where V_RI_ = Reference isodose volume and TV = target volume.

The HI is the ratio between maximum isodose and reference isodose. The formula of HI was shown as Equation [2]. The ideal value is 1, which increases as the plan becomes less homogeneous.

[2] $${\mathrm{HI}}_{\mathrm{RTOG}}=\frac{I_{\max}}{\mathrm{RI}}$$

Where I_max_ = maximum isodose in the target and RI = reference isodose.

The dose gradient (R_x%_) formula is given below:

[3] $$R_{x\%}=\frac{V_{x\%}}{\mathrm{TV}}$$

where V_x%_ = percentage of isodose volume, and TV = target volume.

The pre-optimization, optimization, and re-optimization planning times were compared. The pre-optimization time included OARs contouring and field setup, and the re-optimization time was the time of further optimization and calculation until the plan was satisfied. Average monitor units (MUs) were also evaluated.

### Statistical analysis

The Wilcoxon test was used to compare the differences between the three groups. The differences in the dose coverage, mean dose of the targets, and OARs were compared with a 95% confidence interval. All tests were two-sided. A *p* value of < 0.05 was considered statistically significant. SPSS statistical package (version 17; SPSS Inc., Chicago, IL) was used for all statistical analysis.

## Results

### Target coverage and OAR sparing

Table 1 shows the dosimetric results of targets. For the V_100_, V_107_, D_max_, and D_2_ of the CTV, both MP and RP_F_ groups were significantly better than RP_I_ (*P* < 0.01). MP and RP_F_ in most subjects were not significantly different, except for V_95_. For PTV, the V_100_ was normalized to 95% prescribed dose for all three groups, MP, RP_I_, and RP_F_. MP and RP_F_ groups had significantly better V_107_, D_max_, D_2_, and HI than did the RP_I_ group (*P* < 0.01). The MP group had a worse CI than the other groups. In addition, among 13 compared parameters (Table 1), the RP_I_ had worse results in 84.62% (11/13) parameters compared to the MP and RP_F_ groups, which had the best results in 30.77% (4/13) and 61.53% (8/13) parameters, respectively. The value of HI was the same in the MP and RP_F_ groups.

**TABLE 1.** Dosimetric comparison between manual plans, RapidPlan initial, and RapidPlan final

**Parameters**	**Goals**	**Results**			***P value***		

		**MP**	**RP_I_**	**RP_F_**	**MP *vs.* RP_I_**	**MP *vs.* RP_F_**	**RP_I_ *vs.* RP_F_**
**CTV**							
V_95_ [%]	> 99	99.99 ± 0.03	99.98 ± 0.03	99.97 ± 0.03	0.36	0.03^*^	0.09
V_100_ [%]	> 99	99.20 ± 0.17	98.37 ± 0.33	99.37 ± 0.23	< 0.01^**^	0.07	< 0.01^**^
V_107_ [%]	Minimize	0.62 ± 0.59	2.94 ± 4.33	0.46 ± 0.66	< 0.01^**^	0.16	< 0.01^**^
D_mean_ [Gy]	36	37.23 ± 0.18	37.31 ± 0.21	37.22 ± 0.24	0.07	0.87	0.13
D_max_ [Gy]	Minimize	39.38 ± 0.40	40.38 ± 0.57	39.42 ± 0.41	< 0.01^**^	0.78	< 0.01^**^
D_2_ [%]	< 107	106.12 ± 0.73	106.95 ± 0.96	105.72 ± 0.84	< 0.01^**^	0.19	< 0.01^**^
**PTV**							
V_95_ [%]	> 98	99.68 ± 0.15	99.55 ± 0.23	99.24 ± 0.32	0.03^*^	< 0.01^**^	< 0.01^**^
V_100_ [%]	= 95	95.00 ± 0.00	95.00 ± 0.00	95.00 ± 0.00	-	-	-
V_107_ [%]	Minimize	0.62 ± 0.57	3.01 ± 4.12	0.44 ± 0.61	< 0.01^**^	0.17	< 0.01^**^
D_mean_ [Gy]	36	37.10 ± 0.16	37.22 ± 0.18	37.08 ± 0.20	0.05	0.73	0.01^*^
D_max_ [%]	< 112	109.99 ± 1.17	112.89 ± 1.78	110.17 ± 1.14	< 0.01^**^	0.57	< 0.01^**^
D_2_ [%]	< 107	106.09 ± 0.73	107.00 ± 0.93	105.71 ± 0.80	< 0.01^**^	0.21	< 0.01^**^
CI	1	0.98 ± 0.01	1.01 ± 0.01	1.00 ± 0.01	< 0.01^**^	< 0.01^**^	0.01^*^
HI	1	1.10 ± 0.01	1.13 ± 0.02	1.10 ± 0.01	< 0.01^**^	0.57	< 0.01^**^

CI = conformity index; CTV = clinical target volume; Dx = minimum dose received by the hottest x% volume; HI = homogeneity index; MP = manual plan; PTV = planning target volume; RP_I_ = RapidPlan initial; RP_F_ = RapidPlan final; Vx = volume receiving at least x dose;

* = *P* < 0.05;

** = *P* < 0.01

Furthermore, there were 14 OARs and 20 evaluation parameters for these OARs (Table 2). RP_F_ and RP_I_ had better dosimetric results than MP for the D_mean_ of optic nerves, parotid glands, heart, and esophagus, and D_max_ of eyes (all *P* < 0.05). The RP_F_ group was significantly better than the RP_I_ group in 11 parameters (*P* ≤ 0.01); no parameter in the RP_F_ group was worse than any parameter in the RP_I_ group. RP_F_ had comparable results to the MP group in the other OARs including, brain, brain stem, chiasma, lens, thyroid, lungs, liver, and kidneys. In conclusion, when comparing the three groups, except the heart V_40,_ which was 0% in all these three groups, the MP and RP_I_ groups obtained the worst results in 63.16% (12/19) and 36.84% (7/19) OAR parameters, respectively. On the contrary, the RP_F_ group had 73.68% (14/19) OAR parameters that were superior or equal to the other two groups.

**TABLE 2.** Dosimetric goals and results for organs at risk

**OAR parameters**	**Goals**	**Results**			***P value***		

		**MP**	**RP_I_**	**RP_F_**	**MP *vs.* RP_F_**	**MP *vs.* RP_F_**	**RP_I_ *vs.* RP_I_**
**Brain**							
D_max_ [Gy]	< 60	39.34 ± 0.39	40.24 ± 0.61	39.28 ± 0.37	< 0.01^**^	0.46	< 0.01^**^
**Brain stem**							
D_max_ [Gy]	< 54	38.48 ± 0.41	39.03 ± 0.46	38.51 ± 0.28	< 0.01^**^	0.91	< 0.01^**^
**Chiasm**							
D_mean_ [Gy]	< 50	37.15 ± 0.35	37.10 ± 0.32	36.95 ± 0.32	0.69	0.07	0.06
D_max_ [Gy]	< 55	38.13 ± 0.40	38.73 ± 0.55	38.20 ± 0.26	0.01^*^	0.46	< 0.01^**^
**Optic nerves**							
D_mean_ [Gy]	< 50	27.61 ± 3.40	22.90 ± 2.39	22.42 ± 2.29	< 0.01^**^	< 0.01^**^	0.05
D_max_ [Gy]	< 55	37.13 ± 0.69	36.36 ± 1.72	36.15 ± 1.39	0.13	0.02^*^	0.13
**Eyes**							
D_max_ [Gy]	< 50	25.55 ± 3.57	22.52 ± 3.83	21.60 ± 3.86	0.02^*^	0.01^*^	0.01^*^
**Lens**							
D_max_ [Gy]	< 10	8.40 ± 0.68	8.87 ± 1.00	8.10 ± 0.55	0.11	0.21	< 0.01^**^
**Parotid glands**							
D_mean_ [Gy]	< 25	7.38 ± 2.52	5.16 ± 0.39	4.95 ± 0.39	< 0.01^**^	< 0.01^**^	< 0.01^**^
**Spinal cord**							
D_max_ [Gy]	< 50	38.93 ± 0.51	39.81 ± 0.67	39.04 ± 0.56	< 0.01^**^	< 0.01^**^	< 0.01^**^
**Thyroid**							
D_max_ [Gy]	< 45	17.23 ± 4.04	16.68 ± 2.13	16.38 ± 2.04	0.59	0.36	0.07
**Lungs**							
D_mean_ [Gy]	< 13	4.63 ± 0.30	4.95 ± 0.43	4.63 ± 0.24	0.03^*^	0.73	< 0.01^**^
V_20Gy_ [%]	< 22	0.06 ± 0.11	0.04 ± 0.08	0.03 ± 0.04	0.64	0.44	0.33
V_5Gy_ [%]	< 42	36.48 ± 2.88	42.77 ± 5.62	37.11 ± 2.87	0.01^*^	0.69	< 0.01^**^
**Heart**							
D_mean_ [Gy]	< 10	6.76 ± 1.47	5.53 ± 0.82	5.70 ± 0.93	0.01^*^	0.02^*^	0.33
V_40Gy_ [%]	< 3	0.00 ± 0.00	0.00 ± 0.00	0.00 ± 0.00	-	-	-
V_18Gy_ [%]	< 5	0.04 ± 0.10	0.01 ± 0.03	0.01 ± 0.02	0.31	0.23	0.41
**Esophagus**							
D_mean_ [Gy]	< 34	14.34 ± 1.62	13.23 ± 1.63	13.30 ± 1.74	0.01^*^	< 0.01^**^	0.96
**Liver**							
D_mean_ [Gy]	< 30	4.82 ± 0.94	4.57 ± 0.73	4.45 ± 0.70	0.61	0.33	< 0.01^**^
**Kidneys**							
D_mean_ [Gy]	< 18	2.81 ± 1.17	2.47 ± 0.46	2.38 ± 0.43	0.96	0.73	< 0.01^**^

OAR = organ at risk; MP = manual plan; RP_I_ = RapidPlan initial; RP_F_ = RapidPlan final; Vx = volume receiving at least x dose;

* = *P* < 0.05;

** = *P* < 0.01

Overall, the RP_F_ group achieved superior or equal best results in 71.88% (23/32) of the 32 evaluation parameters of the targets (13) and OARs (19), which excluding the PTV V_100%_ and heart V_40Gy_, because the volumes were the same in all three groups.

In this study, we evaluated the quality of the treatment plans for three groups of 15 patients each. We used 27 parameters to evaluate each plan, for a total of 404 parameters, due to one patient who did not have a thyroid gland. We did not include the parameters CTV V_107%_, CTV D_mean_, CTV D_max_, PTV V_107%_, PTV D_mean_, CI, and HI in the evaluation because they did not have specific goal values. The plan quality pass rate of the MP and RP_F_ groups was 100% (404/404) according to the plan goals of targets and OARs. The RP_I_ group pass rate was 89.36% (361/404). When evaluating the failures of the RP_I_ group, although no patient in the RP_I_ group passed the CTV V_100_ goal of 99%, the minimum and median values of RP_I_ CTV V_100_ were 97.83% and 98.44%, respectively, and both the CTV V_95_ and the PTV V95 of RP_I_ group reached the goals. The pass rates of CTV D_2_, PTV D_max_, and PTV D_2_, for the RP_I_ group, were 66.67% (10/15), 33.33% (5/15), and 66.67% (10/15), respectively. In addition, in the OAR, the lens D_max_ and lungs V_5_ of the RP_I_ group did not meet the goals. The pass rate of the lens D_max_ was 93.33% (14/15) for the RP_I_ group. In one RP_I_ plan, the lens D_max_ was 10.98 Gy > 10 Gy. Lastly, the RP_I_ lungs V_5_ pass rate was 53.33% (8/15).

Table 3 shows the mean dose of the 9 OARs. The highest OARs D_mean_ of the optic nerves, eyes, parotid glands, thyroid, heart, liver, and kidneys; and lens and lungs in these three groups were obtained in the MP group (78%, 7/9) and RP_I_ group (22%, 2/9), respectively. The lowest OARs D_mean_ were mostly in the RP_F_ group (89%, 8/9). Comparing RP_I_ and MP, RP_F_ and RP_I_, and RP_F_ and MP groups, the RP_I_ group significantly reduced the doses of optic nerves, eyes, parotid glands, and heart than the MP group; the RP_F_ group further significantly reduced the doses of eyes, lenses, parotid glands, thyroid, lungs, liver, and kidneys than the RP_I_ group (*P* ≤ 0.05); and RP_F_ significantly reduced the doses of optic nerves, eyes, parotid glands, thyroid, and heart, respectively than the MP group (*P* < 0.05).

**TABLE 3.** The mean dose of the OARs outside the targets contours

**Organ**	**Mean dose**			***P value***		

	**MP**	**RP_I_**	**RP_F_**	**MP *vs.* RP_I_**	**MP *vs.* RP_F_**	**RP_I_ *vs.* RP_F_**
**Optic nerves**	**27.61 ± 3.40**	22.90 ± 2.39	22.42 ± 2.29	< 0.01^**^	< 0.01^**^	0.05
**Eyes**	**12.11 ± 1.99**	10.16 ± 0.55	9.83 ± 0.74	0.01^*^	0.01^*^	0.02^*^
**Lens**	7.09 ± 0.67	**7.21 ± 0.52**	6.78 ± 0.39	0.43	0.16	< 0.01^**^
**Parotid glands**	**7.38 ± 2.52**	5.16 ± 0.39	4.95 ± 0.39	< 0.01^**^	< 0.01^**^	< 0.01^**^
**Thyroid**	**10.41 ± 3.37**	9.00 ± 1.94	8.51 ± 2.07	0.06	0.04^*^	< 0.01^**^
**Lungs**	4.63 ± 0.30	**4.95 ± 0.43**	4.63 ± 0.24	0.03^*^	0.73	< 0.01^**^
**Heart**	**6.76 ± 1.47**	5.53 ± 0.82	5.70 ± 0.93	0.01^*^	0.02^*^	0.33
**Liver**	**4.82 ± 0.94**	4.57 ± 0.73	4.45 ± 0.70	0.61	0.33	< 0.01^**^
**Kidneys**	**2.81 ± 1.17**	2.47 ± 0.46	2.38 ± 0.43	0.96	0.73	< 0.01^**^

Bold type = the highest D_mean_ in the three groups; MP = manual plan; RP_I_ = RapidPlan initial; RP_F_ = RapidPlan final; Underline mark = the lowest D_mean_ in the three groups;

* = *P* < 0.05;

** = *P* < 0.01

In the low-dose region of normal tissue, we employed R_50%_, R_30%_, and R_10%_ as dose gradient indicators. The values for MP, RP_I_, and RP_F_ at R_50%_ were 2.27 ± 0.13, 2.26 ± 0.16, and 2.26 ± 0.14, respectively. For R_30%_, the values were 3.96 ± 0.31, 3.95 ± 0.32, and 3.94 ± 0.37, respectively. The corresponding values for R_10%_ were 10.15 ± 1.93, 10.08 ± 1.69, and 10.00 ± 1.74. There were no statistically significant differences among the three groups (P > 0.05).

**FIGURE 3.**
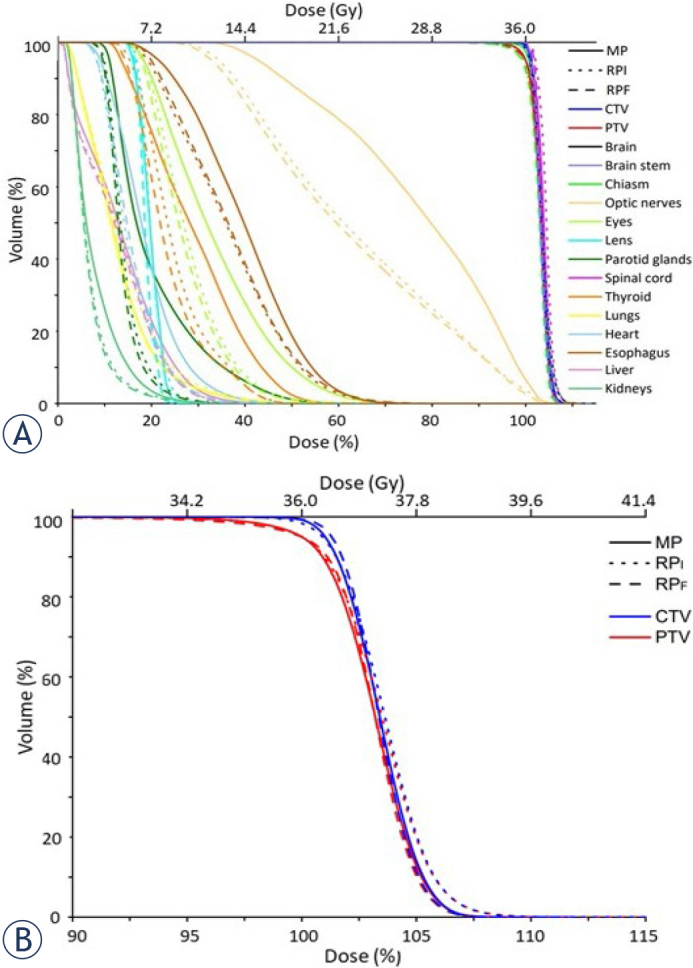
**(A)** Population-averaged dose-volume histogram (DVH) for all organs at risk and targets. **(B)** The population-averaged DVH for targets only. CTV = clinical target volume; MP = manual optimization plan; PTV = planning target volumes; RP_I_ = RapidPlan initial; RP_F_ = final RapidPlan after manual re-optimization

Figure 3A showed the population-averaged DVH of targets and OARs. In the DVH, the doses of optic nerves, eyes, lens, parotid glands, thyroid, liver, and kidneys in RP_F_ or RP_I_ were lower than those in MP. Furthermore, the DVH of RP_F_ OARs was better than those of RP_I_ OARs. Figure 3B shows the targets coverage of CTV and PTV. In the shoulder part of the DVH, with the 95% volume of targets, the MP and RP_I_ groups had the same targets coverage, while the RP_F_ group had a slightly better 95% volume dose coverage than the other two groups. The DVH tail part, the high dose in 5% volume, showed that the RP_I_ had the highest dose in the craniospinal area. The population-averaged DVH showed that the RP_F_ group had the best targets coverage, homogenous targets dose distribution, and OAR dose avoidance among these three groups.

### Treatment planning time

**FIGURE 4.**
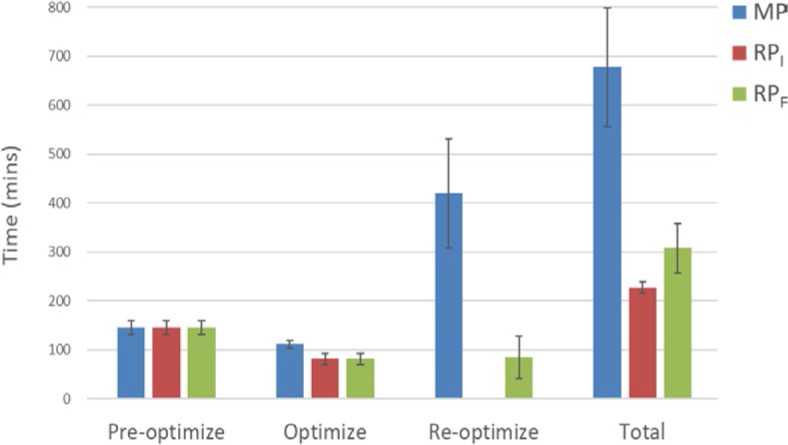
Comparison of the planning time for MP, RP_I_, and RP_F_. The error bar represents one standard deviation. MP = manual optimization plan; RP_I_ = RapidPlan initial; RP_F_ = final RapidPlan after manual re-optimization

The pre-optimization time was the same in all three groups (146 minutes, Figure 4). The optimization process took a significantly longer time in the MP group than in the RP_I_ and RP_F_ groups with 111.45, 81.68, and 81.68 minutes (*P* < 0.05), respectively. The re-optimization time in the MP was significantly longer than in the RP_F_ group (420.36 versus 85.13 minutes, *P* < 0.05). There was no re-optimization in the RP_I_ group. Overall, the entire planning time was longer in the MP group than in the RP_I_ (677.80 versus 227.66 minutes, *P* < 0.05) and RP_F_ (677.80 versus 307.76 minutes, *P* < 0.05) groups. The total planning time-saving rates (saved planning time) of RP_I_ and RP_F_ were 66.41% (450.14 minutes) and 54.59% (370.04 minutes), respectively, compared to the MP group.

### MU comparison

The average MU values with one standard deviation of MP, RP_I_, and RP_F_ groups were 935.24 ± 128.44, 1013.22 ± 114.92, and 1026.46 ± 149.43, respectively, with no significant difference between these three groups (all *P* > 0.05).

## Discussion

Our research discovered that by utilizing 23 plans to develop the KBP model in combination with RP and re-optimization in CSI, we were able to significantly shorten the planning time by half and enhance plan quality.

Incorporating more patients in the model libraries for model training have a possibility to lead to fewer outliers and more consistent plan quality.^15,16,17^ However, the application of the CSI technique in clinical practice is not common in most hospitals. In this study, because CSI treatment is relatively rare, we searched databases covering the previous 6 years and found only 38 CT image sets. The Varian accelerator company recommended a minimum of 20 to 25 treatment plans in training set for a specific target. According to the study by Jim P. Tol *et al.*^18^, Increasing the number of plans used in model training was found to produce comparable results. Based on recommendations, previous experience, and the limited availability of clinical CSI cases, we used 23 plans to complete the model training and compared them with 15 manual plans.

The traditional CSI used patient prone position to reduce the OARs radiation dose via simple two lateral opposed and posterior-anterior (PA) fields. However, this technique can create dose ununiform in the field junction area. The commonly encountered pediatric CSI typically requires two fields and one junction to achieve coverage. This study aims to validate whether KBP can perform effectively in more complex scenarios, utilizing adult CSI as a test case. We used the VMAT technique to disperse the radiation dose in OARs and enhance the homogeneity of the targets dose. The VMAT technique delivers radiation from all angles, which causes it to be attenuated as it passes through the couch. Our medical physicist compensated for this effect by calculating the attenuation of the couch.^19^ Furthermore, cone beam computed tomography ensured an accurate treatment location. Therefore, in this study, all treatment plans were designed using the supine position, which could make patients more comfortable, relaxed, and stable during treatment.^3,20^

Although the plan parameter pass rate of RP_I_ was only 89.36%, the RP_I_ target coverage of minimum CTV V_95_ and PTV V95 values were ≥ 99.90% and ≥ 99.00%, respectively, which were both higher than 95%, the clinical common plan acceptable criteria.^21^ Compared with the traditional 3D-CRT technique, by which the high dose area might receive approximately twice the prescribed dose at the field overlapping sites, the highest PTV D_max_ in RP_I_ was 115.57% which was much lower than the traditional 3D-CRT technique. For OARs, all 14 plans in RP_I_ achieved the goal (< 10 Gy) except for one plan with lens D_max_ 10.98 Gy, which did not reach the goal. Table 2 shows that the heart D_mean_ in RP_I_ was also the lowest of the three groups. Although, Uehara *et al.* reported that KBP was found clinically unacceptable after a single optimization without manual objective constraints in head and neck cancer.^22^ Most studies in the other body sites, such as gynecological, prostate, and rectal cancers, support that the RP plan would be comparable to the manual plan.^23^ In our study, the RP_I_ plans were clinically acceptable for CSI and approved by the physician.

The DVH distribution is one of the vital plan evaluation tools. The DVH of OARs (Figure 3) showed that most of the OARs in the MP group received higher doses than RP_I_ and RP_F_, as shown by the D_mean_ and D_max_ in Table 2. In the target DVH (Figure 3B), the RP_F_ group had better 95% volume dose coverage and better performance at reducing high doses than the other two groups. According to our CI results, there was a minor deviation of the target in the MP group; however, RP_I_ or RP_F_ could have achieved the planning goal. Furthermore, HI values in this study show that MP and RP_F_ groups had better homogeneity than did RP_I_. Previous studies on lung cancer or prostate cancer showed that KBP could reduce the OARs dose^23^; however, target coverage and dose homogeneity of KBP did not always have better results than the manual plan. Our study on CSI showed that RP improved the plan quality of OARs and that additional re-optimization after initial RP could improve the plan quality, as previous studies showed in other cancer sites.^24,25,26,27^

In terms of cardiac doses, all three plans (MP, RP_I_, and RP_F_) exhibited notably low V_40Gy_ and V_18Gy_ values, comfortably below the established cardiac dose constriants. It is pertinent to mention that the mean cardiac dose for RP_I_ was already lower than that for MP. Therefore, the primary focus during the optimization process was not predominantly on further reducing cardiac dose. In the case of RP_I_, the lungs V_5Gy_ value(42.77 ± 5.62%) surpassed the target threshold of 42%. Subsequently, in the ensuing RP_F_ optimization, concerted efforts were undertaken to amplify the reduction of lungs V_5Gy_ values, resulting in a dose shift towards the heart. Nevertheless, from a statistical perspective, the P-value for the comparison between RP_I_ and RP_F_ exceeded 0.05.

In our study, RP_I_ and RP_F_ reduced planning time compared to MP by 66.41% (450.14 minutes) and 54.59% (370.04 minutes), respectively. The result showed that KBP for CSI might save more planning time in complex plans with many OARs than in general cancer sites. Previously, Wells *et al.*^28^ reported that KBP could reduce planning time by approximately 30 minutes per breast cancer patient. Visak *et al.*^29^ reported that all the RP plans required less than 30 minutes of planning time for lung cancer. Masi *et al.*^30^ showed that the time required for the production of the KBP plan was 6–15 minutes, compared to manual planning requiring 30–150 minutes for a commercial TPS and 15–60 minutes after 8 months of commercial TPS usage in prostate cancer. Furthermore, Chatterjee *et al.*^31^ showed that the KBP planning time for the multi-form brain glioblastoma was typically 13 minutes for VMAT, compared to the typical 4 hours for the manual planning method. Amaloo *et al.*^32^ showed that the total planning time was reduced from 120 minutes to 20 minutes in prostate cancer patients. In a study of nasopharyngeal cancer, Chang *et al.*^33^ concluded that the total RP planning time is only about one-fifth that of MP. Similarly, our KBP study for CSI, a very long treatment size from the brain to the lumbosacral area, could effectively reduce the planning time while improving the plan quality, as shown in previous KBP studies for other cancer sites.

## Conclusions

This study used 23 plans to train the KBP CSI model and investigated the difference between MP and RP for the same patients and found that RP plans after re-optimization could halve the planning time and improve plan quality. According to our study result, medical physicists at low CSI patient volume hospitals could efficiently produce CSI plans by the KBP method.
